# Mitochondrial Structure, Function and Dynamics Are Temporally Controlled by c-Myc

**DOI:** 10.1371/journal.pone.0037699

**Published:** 2012-05-21

**Authors:** J. Anthony Graves, Yudong Wang, Sunder Sims-Lucas, Edward Cherok, Kristi Rothermund, Maria F. Branca, Jennifer Elster, Donna Beer-Stolz, Bennett Van Houten, Jerry Vockley, Edward V. Prochownik

**Affiliations:** 1 Division of Hematology/Oncology, Department of Pediatrics, Children's Hospital of Pittsburgh of UPMC, Pittsburgh, Pennsylvania, United States of America; 2 Division of Medical Genetics, Children's Hospital of Pittsburgh of UPMC, Pittsburgh, Pennsylvania, United States of America; 3 Division of Nephrology, Children's Hospital of Pittsburgh of UPMC, Pittsburgh, Pennsylvania, United States of America; 4 Department of Surgery, Children's Hospital of Pittsburgh of UPMC, Pittsburgh, Pennsylvania, United States of America; 5 Department of Cell Biology and Physiology, The University of Pittsburgh, Pittsburgh, Pennsylvania, United States of America; 6 Center for Biological Imaging, The University of Pittsburgh, Pittsburgh, Pennsylvania, United States of America; 7 The University of Pittsburgh Cancer Institute, The University of Pittsburgh, Pittsburgh, Pennsylvania, United States of America; 8 Department of Pharmacology and Chemical Biology, The University of Pittsburgh, Pittsburgh, Pennsylvania, United States of America; 9 Department of Human Genetics, Graduate School of Public Health, The University of Pittsburgh, Pittsburgh, Pennsylvania, United States of America; 10 Department of Microbiology and Molecular Genetics, The University of Pittsburgh, Pittsburgh, Pennsylvania, United States of America; University of Nebraska Medical Center, United States of America

## Abstract

Although the c-Myc (Myc) oncoprotein controls mitochondrial biogenesis and multiple enzymes involved in oxidative phosphorylation (OXPHOS), the coordination of these events and the mechanistic underpinnings of their regulation remain largely unexplored. We show here that re-expression of Myc in *myc−/−* fibroblasts is accompanied by a gradual accumulation of mitochondrial biomass and by increases in membrane polarization and mitochondrial fusion. A correction of OXPHOS deficiency is also seen, although structural abnormalities in electron transport chain complexes (ETC) are not entirely normalized. Conversely, the down-regulation of Myc leads to a gradual decrease in mitochondrial mass and a more rapid loss of fusion and membrane potential. Increases in the levels of proteins specifically involved in mitochondrial fission and fusion support the idea that Myc affects mitochondrial mass by influencing both of these processes, albeit favoring the latter. The ETC defects that persist following Myc restoration may represent metabolic adaptations, as mitochondrial function is re-directed away from producing ATP to providing a source of metabolic precursors demanded by the transformed cell.

## Introduction

As one of the most frequently deregulated oncoproteins in human cancer [1], [2] c-Myc (hereafter, Myc) exerts pleiotropic effects on proliferation, survival, cell cycle, size, differentiation, genomic stability, and metabolism [3], [4], [5], [6]. As might be anticipated for a protein exerting such global influence, Myc regulates a large number of downstream target genes transcribed by RNA polymerases I–III [4], [7], [8], [9], [10], [11]. A significant number of Myc's Pol II-regulated transcripts encode proteins involved in ribosome biosynthesis, aerobic and anaerobic metabolism, and mitochondrial biogenesis [12], [13], [14], [15]. It is believed that the proteins encoded by these genes are needed to sustain the high proliferative demands of transformed cells [16].

The metabolic reprogramming that results from Myc deregulation is exemplified by the “Warburg effect” whereby ATP originating from mitochondrial sources is largely supplanted by that derived from glycolysis, even in oxygen-rich environments [16]. Among the benefits thought to be afforded by the switch to this less efficient mode of energy generation is a redirecting of TCA intermediates away from ATP production and towards the synthesis of lipid, protein and nucleic acid precursors that serve the increased synthetic demands of the rapidly proliferating transformed cell [14], [16], [17], [18]. The resultant increases in mitochondrial biogenesis and metabolism that accompany this reprogramming are at least partly explained by the ability of Myc to regulate the expression of TFAM, a major determinant of mitochondrial DNA replication [12], as well as PGC-1α [19] and PGC-1β [15], which regulate mitochondrial mass and energy metabolism [20].

In addition to increased mitochondrial number, the fusion of pre-existing organelles could provide an independent means of increasing functional efficiency in the face of Myc deregulation. Normally, fusion is thought to allow the mixing and dilution of oxidatively damaged membranes and intra-mitochondrial contents whose excessive accumulation can otherwise lead to the complications such as: the uncoupling of oxidative phosphorylation (OXPHOS), the depletion of ATP pools, and the loss of inner mitochondrial membrane permeability [13], [21]. Fusion might thus complement Myc-mediated *de novo* biosynthesis by minimizing the number of irreversibly damaged mitochondria and thereby prolonging their life spans [22], [23], [24], [25]. This protective function would be particularly valuable given the fact that mitochondria are the major source of the reactive oxygen species (ROS) that are elevated by Myc overexpression [26], [27]. The larger and more efficient mitochondria might also be better able to serve the metabolic needs of the more actively proliferating transformed cell, perhaps in a capacity analogous to that described in hypertrophic cardiac muscle [19], [28].

Fusion, however, is only partially effective at preserving mitochondrial integrity. In the face of overwhelming damage, mitochondria fission produces small, dysfunctional organelles that can be selectively eliminated by the autophagosomal machinery [29]. Fission is also used to reduce mitochondrial mass in the face of rapid reductions in metabolic demands [30]. Thus, both mitochondrial mass and function appear to be highly responsive to the metabolic environment and are coordinately orchestrated by a well-balanced combination of *de novo* synthesis and remodeling via the fission/fusion processes [31], [32].

In the current work, we have investigated the kinetics of mitochondrial assembly and disassembly, along with the mechanisms underlying these processes, by inducing or inactivating Myc in several cell types. We find that conditional Myc depletion is associated with a rapid decline in mitochondrial structural integrity and function, as well as abnormalities in the electron transport chain (ETC) supercomplexes. In contrast, Myc re-expression leads to relatively slower and asymmetric normalization of mitochondria mass and high rates of OXPHOS, despite only partial reversal of ETC complex abnormalities. The coordination of these structural and functional alterations seems to occur in tandem with changes in the levels of several key proteins involved in mitochondrial fission and fusion. These findings shed light on the mechanisms underlying the increases in mitochondrial mass and metabolic reprogramming that accompany Myc deregulation in cancer.

## Results

### Control of mitochondrial structure by Myc

**Figure 1:**
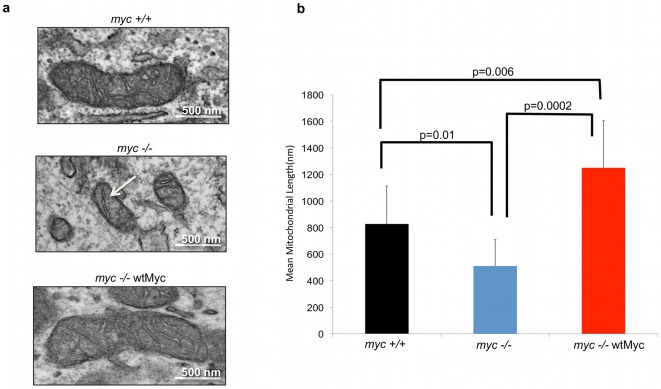
Levels of Myc affect mitochondrial structure in rat fibroblasts. (**a**) *Mitochondrial ultrastructure*. The indicated cell lines were examined by transmission electron microscopy. Representative mitochondria are presented. Note the smaller and cristae-deficient mitochondrion of the *myc−/−* cells (arrow) and the complete restoration of this defect in *myc−/−*wtMyc cells. (**b**) *Mitochondrial length*. Mean mitochondrial length in each cell line was determined by measuring at least 30 individual mitochondria from images of multiple cells obtained by transmission electron microscopy. The student t-test was used to calculate the p-values indicated in the figure.

Initially, we employed three rat fibroblast lines: one with endogenous levels of Myc (*myc+/+*) [33], an isogenic line bearing a homozygous deletion of *myc* (*myc−/−*) [34], and *myc−/−* cells stably transduced with a lentiviral vector encoding wild-type human Myc (*myc−/−*wtMyc cells). Immunoblotting showed that Myc was overexpressed in *myc−/−*wtMyc cells relative to *myc+/+*cells (Figure S1). Differential Myc expression resulted in significant ultrastructural differences as observed by transmission electron microscopy (Figure 1a). Of particular note was that the mitochondria of *myc−/−* cells were not only less abundant and smaller (average length approx. 500 nm) than those of *myc+/+* cells (average length approx. 800 nm), but lacked their more elaborate cristae patterns. Additionally, the mean mitochondrial length in *myc+/+*wtMyc cells was over 50% greater than that of *myc+/+* cells (approx. 1300 nm, Figure 1b). From these studies, we conclude that endogenous Myc plays a role in mitochondrial size and structural integrity.

In order to study the Myc-induced changes in mitochondria as a function of time, we next utilized *myc−/−* cells transduced with a retrovirally-encoded, 4-hydroxytamoxifen (4-HT)-responsive Myc-estrogen receptor (MycER) fusion protein [35], which we hereafter refer to as MycER cells. In the absence of 4-HT, the MycER fusion protein is sequestered in the cytoplasm [35], [36]. After binding 4-HT, MycER undergoes a conformational change, is shuttled to the nucleus, and becomes transcriptionally active.

**Figure 2:**
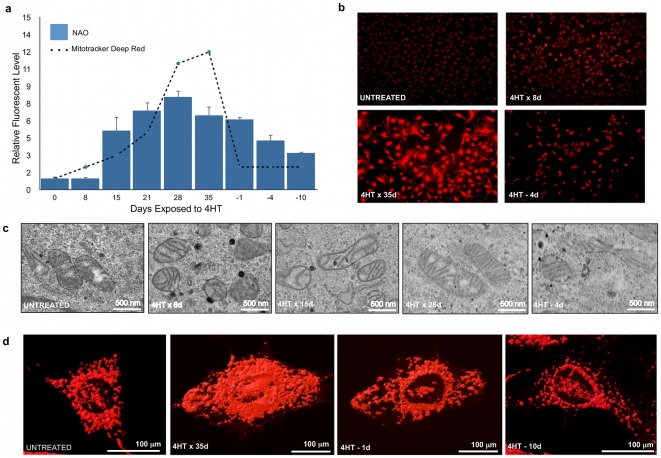
Temporal changes in mitochondrial density and structure in response to Myc. (**a**) *Mitochondrial Mass*. MycER cells were exposed to 4-HT for up to 35 d at which point 4-HT was removed. At the indicated times, cells were stained with NAO and MitoTracker Deep Red and analyzed by flow cytometry. Values are expressed relative to control cells not exposed to 4-HT, which were arbitrarily set to a value of one. (**b**) *Mitochondrial mass by microscopy*. MycER cells stained with MitoTracker Red were observed by epifluorescence microscopy at low power. The intensity of staining is consistent with the finding from flow cytometry. (**c**) *Mitochondrial ultrastructure*. MycER cells were exposed to 4-HT for the indicated times and observed by transmission electron microscopy. Representative mitochondria from each time point are depicted. Note that the untreated cells had small, cristae-deficient mitochondrion similar to those of the *myc−/−* cells. After several weeks of Myc induction the mitochondria took on a more normal and mature appearance. The size of the mitochondria began to decrease as 4-HT was removed from the media. The addition of 4-HT to cells lacking the MycER construct had no effect on any of the above-measured mitochondrial properties (not shown). (**d**) *Mitochondrial shape*. MycER cells were observed under live confocal microscopy either prior to or following the addition of 4-HT for 35 days. 4-HT was then removed and the cells were imaged again 1 day or 10 days later. Mitochondria were stained with MitoTracker Deep Red and 3-dimensional reconstructions of the cells were performed from a series of z-stacks. Shown are representative images with a space-filling overlay to emphasize the mitochondria. Note the loss of total mitochondria and the decrease in interconnectivity upon removal of 4-HT. Additional cells can be seen in a video detailing the three-dimensional structure (Video S1).

We used 10-n-nonyl-acridinium-orange-chloride (NAO) to quantify mitochondrial mass in MycER cells in the logarithmic growth phase. Following the addition of 4-HT, an increase in NAO staining was noted over the ensuing three to four weeks. Upon reaching this stage of “chronic” exposure (i.e. >28 days), 4-HT was removed from the medium and cells were again stained with NAO, which revealed that the previously observed increase in mitochondrial mass was reversible, with NAO staining patterns gradually decreasing, but remaining at elevated levels for as long as 10 days (Figure 2a).

The same cells were stained with MitoTracker® Deep Red which also measures mitochondrial mass, but does so in a manner that is more dependent on the maintenance of mitochondrial membrane potential ([25], [37], [38]; Figure 2a). We noted a somewhat more gradual increase in this signal following the addition of 4-HT, but a much more rapid decline upon its removal. A similar pattern was seen in the corresponding epifluorescence images of the cells (Figure 2b), suggesting that the maintenance of mitochondrial membrane potential is more dependent on ongoing Myc activity than is mitochondrial mass. In the absence of 4-HT, MycER cells were similar in appearance to *myc−/−* cells with regard to mitochondrial morphology (Figure 2c). Upon activation of Myc activity, the normalization of mitochondrial length and cristae patterns appeared to occur somewhat more rapidly than did the normalization of total mitochondrial mass. Similarly, these properties were also lost more rapidly than mass upon removal of 4-HT.

Mitochondria are highly interconnected in a manner that often correlates with their mass and function [39]. To determine whether this was the case with the rat fibroblasts being studied here, we generated space filling overlays over three-dimensional reconstructions from serial confocal images obtained on MitoTracker®-stained MycER cells exposed to 4-HT for differing periods of time. Both untreated cells and those from which 4-HT had been removed, showed the reduced mitochondrial mass and loss of connectivity previously noted for *myc−/−* cells. (Figures 2d and Video S1). In contrast, chronic 4-HT treatment was associated with a dense and highly interconnected mitochondrial network. We also noted a tendency for the mitochondria of these latter cells to be highly concentrated in a peri-nuclear pattern. The removal of 4-HT was associated with a selective migration of mitochondria away from nuclei and a more dispersed distribution.

From the above studies, we conclude that mitochondrial mass responds gradually to Myc induction and withdrawal, whereas changes in membrane potential and interconnectivity are more rapid, particularly following 4-HT removal. Finally, a preferential re-localization of mitochondria to sites immediately adjacent to and/or connected with the nuclear membrane occurs following Myc induction.

### Control of mitochondrial function by Myc

**Figure 3:**
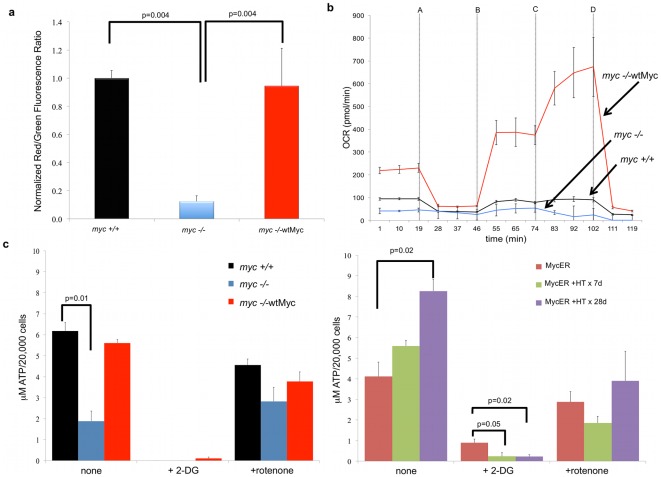
Mitochondrial function. (**a**) *Mitochondrial membrane potential*. Cells were stained with JC-1 and analyzed by flow cytometry. The degree of mitochondrial membrane depolarization was measured by a decrease in the red/green fluorescence ratio. Values are expressed relative to those of *myc+/+* cells, which were arbitrarily set to one. (**b**) *OXPHOS*. A Seahorse Bioscience XF24 Extracellular Flux Analyzer was used for the real-time determination of the oxygen consumption rate (OCR). Each inhibitor was injected at the times indicated by the arrows (injections: A-oligomycin, B-FCCP, C-2-DG, D-rotenone). A typical experiment, performed in triplicate wells is shown. The experiment was repeated on at least three occasions in replicates of four with similar results. When compared to *myc−/−* cells, the p values for the basal OCR for *myc+/+* cells = 0.000004 and for *myc−/−*wtMyc cells = 0.000001. (**c**) *Steady-state ATP levels*. The steady-state levels of ATP were measured for the *myc+/+*, *myc−/−*, and *myc−/−*wtMyc fibroblasts (left) and in MycER cells (right). The assays were performed both in the absence and presence of inhibitors of glycolysis (2-DG) and OXPHOS (rotenone) inhibitors.

The previous studies suggested that Myc-associated changes in mitochondrial mass and structure might also be accompanied by altered mitochondrial function, particularly in light of the rapid decline in MitoTracker Deep Red staining (Figure 2a), which is known to be partially dependent on membrane polarization [40]. To investigate this further, we first compared mitochondrial membrane polarization in living *myc−/−*, *myc+/+*, and *myc−/−*wtMyc cells using the JC-1 dye [41]. We measured the ratio of red-shifted JC-1 aggregates, whose formation is favored under conditions of high membrane potential, and green-shifted monomers, which tend to predominate under conditions of low membrane potential [41]. As seen in Figure 3a, the structurally abnormal mitochondria of *myc−/−* cells showed a nearly ten-fold reduction in membrane polarization relative to that of *myc+/+* and *myc−/−*wtMyc cells, with the latter showing no evidence of hyperpolarization as has been reported to occur in activated T-cells [42].

Having established that the enforced expression of Myc restored mitochondrial membrane potential, we next asked whether basal levels of oxidative phosphorylation (OXPHOS) were affected. We have previously shown that another rat fibroblast line, Rat1a, is more reliant on glycolysis than OXPHOS for its basal energy requirements but that Myc over-expression significantly increases the rates of both [43]. We used the XF24 Extracellular Flux Analyzer to measure oxygen consumption rate (OCR), which serves as a reliable surrogate for OXPHOS [44]. Our findings in *myc−/−*, *myc+/+* and *myc−/−*wtMyc cells were consistent with our previous results. As seen in Figure 3b, basal OCR, as measured between 0 and 19 min. was approximately two-fold higher in *myc+/+* cells than in *myc−/−* cells and an additional 2–2.5-fold higher in *myc−/−*wtMyc cells; thus, OCR was largely correlated with the degree of Myc expression and/or de-regulation.

The cell lines were then sequentially exposed to four metabolic inhibitors to provide a profile of bio-energetic capacity. The first inhibitor, oligomycin (Figure 3b, addition A at 19 minutes), blocks the ATP synthase activity of Complex V and led to the expected cessation of respiration in all cell lines. Cyanide *p*-trifluoromethoxy-phenylhydrazone (FCCP; Figure 3b addition B at 46 minutes) was next injected to promiscuously restore proton flow. The increase in FCCP-mediated OCR provided a measurement of the maximal respiratory rate of cells, which was significantly higher in *myc−/−*wtMyc cells. The metabolic response seen between the addition of FCCP and 2-deoxy-D-glucose (2-DG), which inhibits glycolysis (addition C at 74 minutes), was representative of the so-called “spare respiration capacity (sRC)” [45], [46]. *myc−/−* cells had the lowest sRC, displaying approximately 30% lower levels than *myc+/+* cells, whereas sRC was 10.6-fold higher in *myc−/−*wtMyc cells. Following the addition of 2-DG, *myc−/−*wtMyc cells displayed a further increase in OCR that, in combination with the sRC, represents the total reserve respiratory capacity [45], [46], which was 20-fold higher than the level observed in *myc−/−* cells. Finally, the injection of rotenone (injection D at 102 minutes), which inhibits Complex I, resulted in the complete cessation of electron flow and oxygen consumption in all cell lines. Taken together, these data show that Myc increases both actual baseline OXPHOS as well as the potential to achieve higher levels, in the face of otherwise normal mitochondrial mass.

We next investigated the relationship between OXPHOS and ATP levels in the above cell lines. We found that *myc+/+* cells and *myc−/−*wtMyc cells each contained about three-fold higher steady-state levels of ATP than *myc−/−* cells (Figure 3c). The inhibition of glycolysis by 2-DG was associated with a nearly complete loss of net ATP levels whereas the inhibition of Complex I by rotenone was associated with much more modest decreases. These results suggested that, despite overall higher basal levels of OXPHOS and respiratory reserve capacity of *myc−/−*wtMyc cells, the majority of their ATP supply is derived from glycolysis. This was confirmed by concomitant measurements of the extracellular acidification rate (ECAR) (Figure S2). These results, which measure the increased acidity of the medium as glucose is converted to lactate, showed that ECAR also correlated with Myc expression and were consistent with previous findings that constitutive ectopic Myc expression in Rat1a fibroblasts is associated with a high rate of glycolysis [43]. Relative to *myc−/−* cells, *myc−/−*wtMyc cells showed approximately three-fold higher levels of ECAR (Figure S2). These results were largely reproduced in MycER cells where net ATP content was observed to increase as a function of time of Myc induction and where ATP production was completely abrogated by 2-DG (Figure 3c).

The finding that *myc+/+* cells and *myc−/−*wtMyc cells contained equivalent ATP levels seemed at first to be inconsistent with their different rates of glycolysis and OXPHOS. We therefore measured ATP half-life in each cell line and showed it to be shorter in *myc−/−*wtMyc cells (2.6±1.0 minutes) than in *myc+/+* cells (3.6±0.8 minutes; Figure S3; p = 0.02). Taken together, these results suggest that the apparent discrepancy between ATP levels and rates of OXPHOS in *myc+/+* and *myc−/−*wtMyc cells can likely be explained by higher rates of ATP turnover by the latter cells.

**Figure 4:**
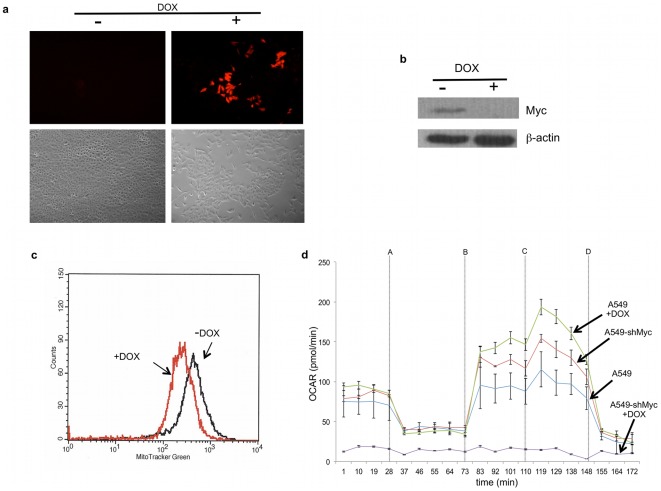
Mitochondrial structure and function in human A549 cells. (**a**) *Effect of Myc shRNA induction*. A549 cells expressing doxycycline (DOX)-inducible Myc shRNA were exposed to DOX for 4 days after being grown to 50–75% confluency. The upper right panel shows the signal from the co-expressed RFP. The upper left panel shows the same cells grown in the absence of DOX. The lower panels represent changes in growth as a result of DOX exposure following the plating of equal numbers of cells and a subsequent four day period of growth +/−4-HT. (**b**) *Endogenous Myc protein levels*. Immunoblotting for Myc protein in control, non-DOX-treated cells or those exposed to DOX for four days. β-actin was used as a loading control. (**c**) *Mitochondrial mass*. Cells were exposed to DOX for four days and stained with MitoTracker Green. (**d**) OXPHOS. A typical experiment as performed on the Seahorse is shown for cells grown in the presence and absence of DOX for four days. Included are control A549 transduced with a lentiviral vector expressing a scrambled shRNA sequence and which do not display any changes in OXPHOS when exposed to DOX.

Finally, we asked whether the effects of deliberately enforced Myc expression on mitochondrial structure and function could be observed in cells in which high endogenous Myc levels were a natural consequence of the transformed state. We therefore repeated several of the above studies in human A549 non-small cell lung cancer cells stably transduced with a lentiviral vector containing a doxycycline (DOX)-inducible Myc-targeted shRNA (A549-shMyc cells). This vector also expressed red fluorescent protein, which permitted the convenient indirect monitoring of shRNA induction. As seen in Figure 4a, a four day DOX treatment of A549-shMyc cells led to the expression of red fluorescent protein, the abrupt cessation of proliferation, and a more flattened cellular morphology. Evaluation of these cells by immunoblotting showed these features to be associated with a nearly complete loss of endogenous Myc expression and a subsequent two-fold reduction in MitoTracker® Green staining (Figure 4b and c). Metabolic analysis of these cells also revealed baseline levels of OXPHOS of DOX-treated A549-shMyc cells to be more than ten-fold reduced relative to non-DOX-treated cells (Figure 4d). Similar to what had been observed in *myc−/−* cells, DOX-treated A549-shMyc cells displayed relatively low basal OXPHOS with little sRC, whereas cells grown in the absence of DOX showed higher levels of both basal OXPHOS and sRC. Collectively, these data indicate that Myc has a positive effect on mitochondrial mass and function in both rat and human cells.

### Regulation of mitochondrial ETC complexes by Myc

**Figure 5:**
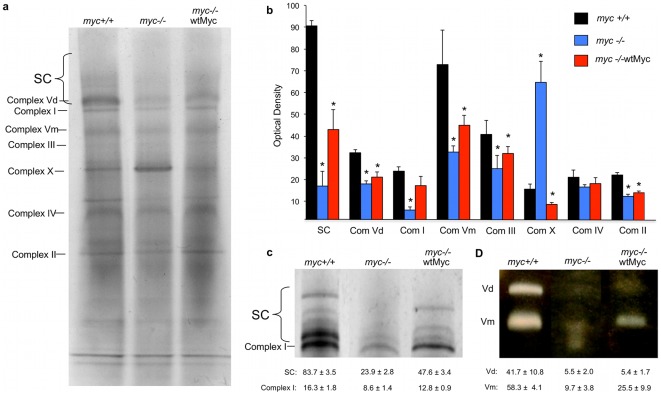
Electron Transport Chain complexes. (**a**) *Blue native gel electrophoresis*. A representative blue native gel is depicted. (**b**) *Quantitation of complex formation*. The histogram is representative of the optical density of Coomassie blue staining for at least three separate experiments. In comparison to the *myc+/+* cells, p values were generated and the strains with values <0.05 are indicated with an asterisk. (**c**) *Complex I in situ gel activity*. Enzymatic activity staining was performed for Complex I. Depicted is a representative experiment. Note that Complex I activity could be detected in the previously identified Complex I band as well as the bands that represent higher order supercomplexes (SC). Total activity of Complex I+SC was calculated for *myc+/+* cells and arbitrarily set to 100%. (**d**) *Complex V in situ gel activity*. Enzymatic activity staining was performed for Complex V. Shown is a representative experiment. Total activity of Complex V (represented as the sum of individual Vm and Vd activities) was calculated for *myc+/+* cells and arbitrarily set to 100%.

The mitochondrial ETC consists of four major protein complexes that mediate a series of redox reactions involving electron transfer from donors (NADH, succinate and fatty acid oxidation products directly through coenzyme Q) to electronegative acceptor molecules terminating with the reduction of oxygen to water [47]. The energy generated during this step-wise process is used to pump protons from the mitochondrial matrix to the inter-membrane space, thereby establishing an electrochemical proton gradient, which ultimately powers the conversion of ADP to ATP via ATP synthase (Complex V). Due to the large differences in the observed rates of OXPHOS and ATP biosynthesis in *myc+/+*, *myc−/−*, and *myc−/−*wtMyc cells (Figure 3), we compared the relative levels of their respective ETC complexes using blue native gel electrophoresis (BNGE) and scanning densitometry (Figure 5a and b). The identification of each complex was further confirmed by silver staining of SDS-PAGE gels to confirm the identity of individual subunits (not shown) and by *in situ* gel activity stains (see below) [48].

Relative to *myc+/+* cells, *myc−/−* cells showed decreases of several respiratory complexes, most notably Complex I, whose levels were reduced approximately four-fold (Figure 5b). Less pronounced reductions were also noted in Complexes II, III, and monomeric Complex V (Vm). These results stand in contrast to the minimal change in Complex IV between these two cell lines. Mitochondria from *myc−/−* cells also contained an unidentified complex (“Complex X”; Figure 5a) that was increased more than four-fold relative to the other two cell types. Preliminary analysis of Complex X by mass spectroscopy indicates that it contains some of the subunits from Complexes II–V, amongst other proteins.

Some ETC complexes are also found in higher order structures, either as homodimers, or in association with other complexes. For example, Complex V occurs in both monomeric (Vm) and dimeric (Vd) forms, with the latter having been proposed to be more enzymatically efficient [49]. Similarly, all individual complexes can associate in various combinations to form more efficient supercomplexes (SCs), such as those comprised of Complexes I+III+IV [50]. These higher order structures were also depleted in *myc−/−* cells as evidenced by the approximately two-fold and five-fold decreases in Complex Vd and SCs, respectively (Figures 5b and 5c).

Given the restoration of mitochondrial mass and OXPHOS in *myc−/−*wtMyc cells, it was expected that they would have similarly normal ETC complexes; rather, they showed only a partial restoration of ETC complexes. Specifically, these cells displayed levels of complexes I, II, Vm, Vd, and SCs that were intermediate to those of *myc−/−* and *myc+/+* cells (Figure 5a and b). Thus, the restoration of Myc by its enforced over-expression only partially corrected the ETC complex abnormalities seen in *myc−/−* cells.

Next, we studied the enzymatic activities of Complexes I and V using previously described *in situ* activity stains [48], [51], [52]. These studies showed that Complex I activity in *myc+/+* cells resided in both the Complex I and SC bands and was decreased by nearly two-thirds in *myc−/−* cells (Figure 5c). Overall, total Complex I activity was only partially restored in *myc−/−*wtMyc cells (Figure 5c).

Similar *in situ* measurements of Complex V ATP synthase activity showed an even more profound reduction in *myc−/−* cells than indicated by the total protein levels depicted in Figure 5b. In this assay, total Complex V activity averaged only 15% of that measured in *myc+/+* cells, with Vm and Vd activities being reduced comparably (Figure 5d). As had been seen with Complex I, the re-expression of Myc in *myc−/−*wtMyc cells only partially restored Complex V activity, with virtually the entire correction occurring at the level of Vm. Overall, these results clearly show that Myc overexpression does not restore either normal respiratory chain function or structure despite normalizations of mitochondrial size, mass and ATP content.

### Regulation of mitochondrial fusion and fission proteins by Myc

Mitochondria are dynamic and interconnected organelles whose overall structural and functional integrity are maintained by a balance between fusion and fission [24], [25], [32]. Because the smaller and poorly communicating mitochondria of *myc−/−* cells could reflect alterations in either or both of these pathways, we examined several key fusion and fission proteins. Those in the former category included the inner membrane-embedded GTPase, Opa1, which plays important roles in the maintenance of mitochondrial shape and cristae formation [32] and the mitofusins Mfn1 and Mfn2 [53], which interact with each other as well as Opa1, and positively regulate membrane potential, metabolism and survival [22], [24], [25], [32], [54], [55], [56]. Proteins in the latter category included the dynamin-like protein Dlp1 and Fis1, which are believed to trigger fission as a result of their interaction on the outer mitochondrial surface [57], [58].

**Figure 6:**
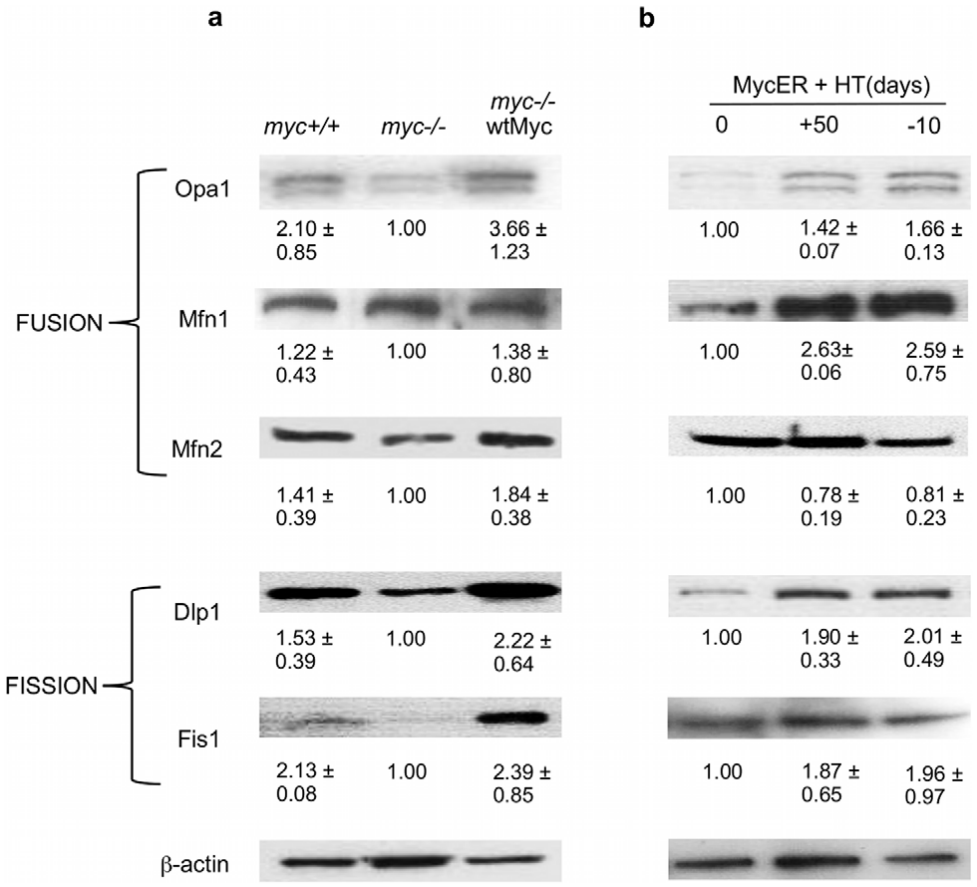
Western analysis of mitochondrial fusion and fission proteins. Western analysis was performed on whole cell lysates from (**a**) *myc+/+*, *myc−/−* and *myc−/−*wtMyc cells and from (**b**) Myc ER cells exposed to 4-HT for the indicated times. β-actin is used as a loading control during each experiment. Densitometry was performed and the values were used to calculate a ratio of the protein expression relative to the level of β-actin. This ratio was normalized and the value for *myc−/−* for (a) and untreated MycER cells for (b) was set to 1. Experiments were repeated a minimum of three times and the standard error between the experiments is shown.

Western analysis of the above cell lines showed both fusion and fission proteins to be expressed at somewhat higher levels in *myc+/+* cells when compared to *myc−/−* cells (Figure 6a). This ranged from a minimal increase in levels of Mfn1 to larger increases in the levels of Fis1 and Opa1. Although our findings were mostly similar in *myc−/−*wtMyc cells, Opa1 and Dlp1 levels generally exceeded those of *myc+/+* cells. These results were largely confirmed in MycER cells where long-term activation of MycER by 4-HT was again associated with variable increases in most of the proteins, with the largest and most significant increase observed for Mfn1, whose level increased more than two-fold when compared to untreated *myc−/−*wtMyc cells (Figure 6b). Surprisingly, there was no decline in fission or fusion protein levels upon 4-HT removal, despite the previously observed loss of mitochondrial mass (Figure 2a).

**Figure 7:**
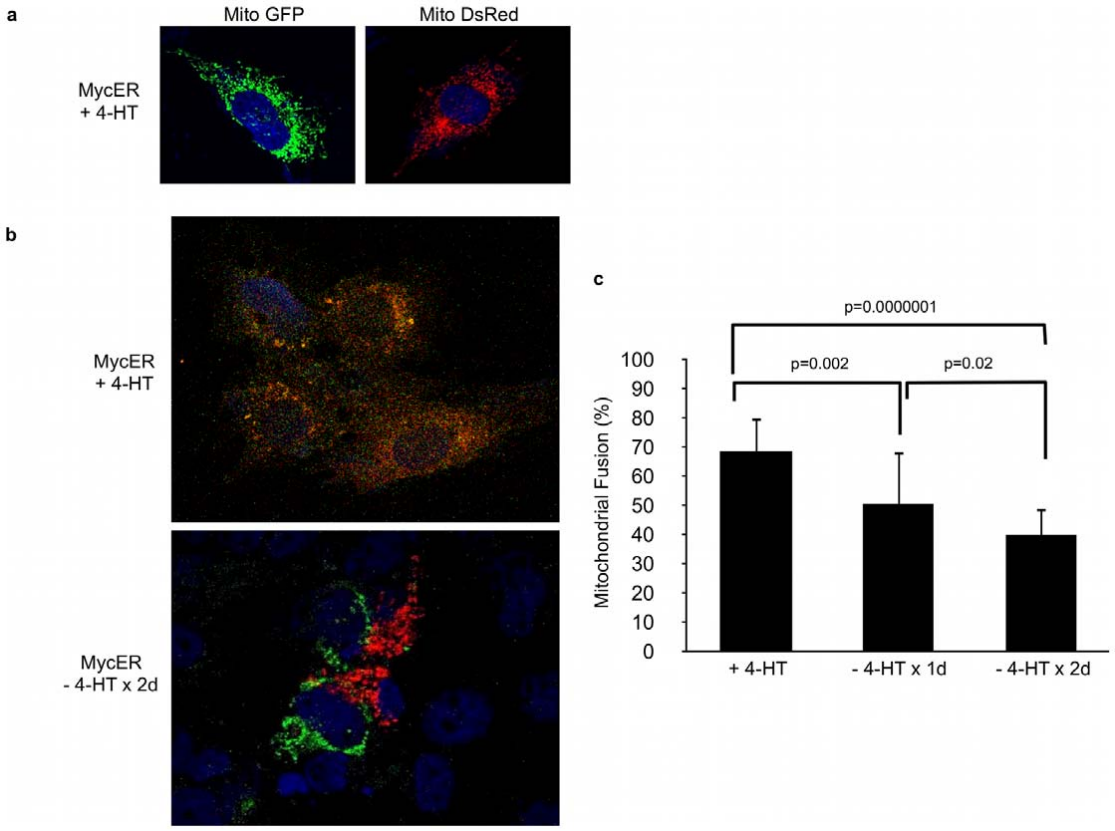
PEG Fusion Assay. (**a**) MycER cells exposed to 4-HT chronically were stably transduced with lentiviruses encoding mitochondrially targeted DsRed or GFP. Photos show representative cells with mitochondrially-targeted fluorescent proteins. (**b**). Cells were co-plated in the presence or absence of 4-HT and were treated with PEG following incubation with CHX. Cells were fixed stained with DAPI and heterokaryons were identified and imaged by confocal fluorescent microscopy. (**c**) The degree of mitochondrial fusion was calculated by determining the percentage of co-localized fluorescent signals with the use of the CoLocalizer Pro software package. Cells from which 4-HT was removed from the media for one or two days were compared to cells constantly maintained in 4-HT.

Given the foregoing results, as well as our evidence indicating that loss of Myc decreased the connections between mitochondria (Figure 2d), we next quantified the amount of fusion activity in MycER cells (Figure 7). Cells that were maintained in 4-HT for over four weeks were stably transduced with lentiviral vectors expressing GFP and DsRed proteins that were targeted to mitochondria by virtue of being fused to the mitochondrial localization signal for subunit VIII of cytochrome c oxidase (Figure 7a). These cells were then co-plated and either maintained in 4-HT or had the inducing agent removed from the medium 24 or 48 hours prior to inducing plasma membrane fusion with polyethylene glycol as previously described [25], [59]. The degree of fusion was measured by quantifying GFP and DsRed co-localization in the resulting heterokaryons (Figure 7b). MycER cells maintained in 4-HT displayed approximately 70% fusion of mitochondria, within two hours of PEG fusion, whereas cells from which 4HT had been removed showed a significantly reduced degree of fusion (Figure 7c). Taken together, these data indicate that mitochondrial biogenesis in response to Myc induction is associated with a coordinated and complex alteration of both fusion and fission proteins, which ultimately favors the former activity. Moreover, and similar to the changes seen in mitochondrial mass and polarization (Figure 2a), this process is rapidly responsive to changes in Myc levels.

## Discussion

Previous studies have shown that Myc regulates mitochondrial biogenesis and that many enzymes of the OXPHOS and glycolytic pathways are under direct Myc control [12], [15], [19]. Precisely how Myc coordinates these activities or other aspects of mitochondrial structure and function remains poorly understood. We have shown here that changes in mitochondrial structure and function are temporally regulated in a highly controlled manner in response to changes in Myc levels. These changes are not symmetrical but instead occur more rapidly in response to Myc's inactivation than to its activation. Most notably, membrane potential and interconnectivity are lost much more rapidly than mitochondrial mass upon Myc inactivation (Figure 2a). Although we do not understand the basis for this temporal asymmetry, it is tempting to speculate that Myc is primarily responsible for maintaining mitochondrial membrane potential and may do so by increasing membrane fusion. We are currently conducting studies to address this phenomenon. Furthermore, the relatively slow response of mitochondria biogenesis to Myc induction relative to other features of the transformed state suggests that changes in metabolism are not necessary for transformation *per se*, but rather serve to provide for the more robust proliferative demands of transformed cells [17], [18], [60]. These results are in keeping with our recent finding that a point mutation in the transactivation domain of Myc (Q131R) retains potent transforming and tumorigenic competence despite being significantly impaired in its ability to up-regulate both glycolysis and OXPHOS [43].

Among the more notable features of *myc−/−* cells was the relative paucity of mitochondria in the perinuclear region (Figure 2d). This localization appears to be an on-going, active process as mitochondria at these sites were preferentially lost in MycER cells following 4-HT withdrawal. Close contact between mitochondria and the nuclear membrane has been proposed to facilitate the transfer of ATP or metabolic precursors to sites of particularly intense local utilization [61]. Similar perinuclear clustering of mitochondria has previously been described in cells that overexpress Mfn1 [62]. Such selective spatial organization is also seen in striated muscle where mitochondria are in close proximity to the sarcoplasmic reticulum and likely play a role in Ca^2+^ buffering [63], [64]. Whether the intimate and apparently fluid association between mitochondria and nuclei described here also has distinct functional consequences remains to be determined. Regardless of the differences of these mitochondrial subsets, the rapidity with which their spatial reorganization occurs in response to abrupt changes in Myc levels shows a close correlation with the structural and metabolic fluctuations that occur in parallel.

Overexpression of Myc in the *myc−/−* background restored mitochondrial mass and volume to wild-type levels, while simultaneously giving rise to mitochondria that were longer than those observed in *myc+/+* cells (Figure 1). We speculate that these longer mitochondria may be a direct result of increased fusion activity. ETC complexes also remained quite abnormal in *myc−/−*wtMyc cells (Figure 5); thus, the excessive OXPHOS potential of *myc−/−*wtMyc cells (Figure 3b) cannot be explained by proportionate changes in ETC complexes. These cells do possess a higher glycolytic rate than the other cells (Figure S2), which is consistent with our previous findings as well as with reports that Myc is a major regulator of the Warburg effect [16], [43], [65]; therefore, much of the energy requirement of these cells appears to be met by ATP generated via aerobic glycolysis (Figure 3). That certain ETC complexes and supercomplexes, and their activities, remained abnormal in *myc−/−*wtMyc cells may therefore be one of the factors leading to the up-regulation of glycolysis. This may also underlie the well-known tendency of Myc over-expression to induce high levels of reactive oxygen species [27] that might be facilitated by structural and/or functional ETC abnormalities leading to an increase leak of electrons, particularly at Complex I [66]. Finally, the ETC abnormalities we have observed may represent a metabolic adaptation that reflects a shift from the production of ATP to the production of the lipid, protein and nucleic acid intermediates needed to meet the higher proliferative demands imposed as a result of Myc deregulation.

The mechanism underlying the changes reported here appears to involve alterations in the levels and the activities of proteins that coordinate mitochondrial fission and fusion. That both types of proteins respond positively to Myc was unexpected and does not allow for a simple model of how, collectively, they preside over increases in mitochondrial mass, polarity and fusion. The degree to which deviations in the normal ratios of these proteins in response to Myc deregulation contribute to mitochondrial structural and functional abnormalities is also a question for future work.

In addition to regulating mitochondrial dynamics, fusion is necessary to maintain structural and functional homogeneity, to preserve mitochondrial genome integrity, and to ensure the proper balance between energy generation and cellular mass [22], [24], [25], [32], [56]. Higher levels of fission proteins might be needed to maintain homeostasis of the greatly increased mitochondrial mass in Myc overexpressing cells as well as to provide a means for the more rapid elimination of oxidatively damaged organelles that are generated as a result of high levels of reactive oxygen species induced by Myc [26], [27]. Our results suggest that the increased mitochondrial mass in response to Myc induction may be associated with high levels of organelle turnover, thus necessitating higher levels of both fission and fusion proteins.

In conclusion, we propose that Myc, which is among the first genes activated in response to a broad array of growth factors, serves to link the proliferative signals mediated by these factors with mitochondrial biogenesis and function via the control of fission and fusion processes [32]. That Myc appears capable of independently controlling both of these arms, coupled with its rapid induction by growth factors and its extremely short half-life, provides the means by which mitochondrial supply and cellular energy generation can be fine tuned to meet cellular proliferative demands.

## Materials and Methods

### Cell culture and virus infections

The generation of *myc+/+* and *myc−/−* rat fibroblasts has been previously described [33], [34]. *myc−/−*wtMyc cells were generated by stable transduction with a lentiviral vector expressing a full-length human wtMyc as previously described [43], and cells were selected for blasticidin resistance. MycER cells were also derived from *myc−/−* cells by transduction with the pBabePuro-MycER retroviral vector and selection in puromycin [35]. To activate MycER, 4-HT (Sigma-Aldrich, St. Louis, MO) was added to a final concentration of 250 nM and the media was replaced every 48–72 hours for all experiments.

DsRed and GFP, both fused to the mitochondrial localization signal of subunit VIII of cytochrome c oxidase (pMito-DsRed and pMito-GFP, Clontech, Mountain View CA) were amplified using the polymerase chain reaction and directionally subcloned into the pLenti6/V5 lentiviral vector as recommended by the supplier (Invitrogen, Carlsbad, CA). They were packaged into infectious viral particles in 293FT cells and transduced into recipient cells followed by selection in blasticidin. A549-shRNA cells were derived from the A549 non-small cell lung cancer cell line by transduction with a pTRIPZ lentiviral vector (Open Biosystems, Huntsville, AL) encoding a DOX-regulatable bi-cistronic transcript for RFP and a shRNA directed against human Myc. Control cells were transduced with the same vector encoding a scrambled shRNA. Stably transfected cells were selected in puromycin and those expressing the highest levels of RFP following overnight exposure to DOX (1 µg/ml) were further selected by fluorescence-activated cell sorting. The cells were then expanded in DOX-free medium. All cell lines were propagated in Dulbecco's-modified minimal essential medium (D-MEM) containing 10% fetal bovine serum, glutamine, and penicillin plus streptomycin as previously described [67], [68]. The working concentration of DOX was 5 µg/ml and the media was replaced every 48 hr. All retroviral and lentiviral work was approved by the University of Pittsburgh Institutional Biosafety Committee and was performed under BSL2+ conditions.

### Fluorescence microscopy

Low power (20×) fluorescent images were obtained using a Zeiss Axiovert fluorescent microscope by direct observation of cells grown on tissue culture dishes and maintained at 37°C and 5% CO_2_. High power (60×) images of cells grown to 50% confluency on glass bottom 6-well tissue culture plates were obtained on a Zeiss LSM710 confocal microscope at 0.5 µm intervals. Observation of mitochondria was possible following staining with MitoTracker® Deep Red FM as described below. The confocal stacks of the mitochondria were uploaded into Imaris (Bitplane Scientific Software) and a 3D reconstruction of the cells was obtained. A surface was then placed over the MitoTracker positive stain using the Imaris software. 3D movies of the reconstructed mitochondria were then generated.

### Electron microscopy

Cells were grown to confluency in 12-well tissue culture dishes and fixed with 2.5% glutaraldehyde. Samples were prepared as previously described [69] and subsequently were photographed using a JEM 1,210 transmission electron microscope (JEOL, Peabody, MA) equipped with a CCD camera (Advanced Microscopy Techniques Corp., Danvers, MA) at 80 kV.

### Quantitation of mitochondrial mass and membrane potential

Mitochondrial mass was measured by staining live cells with NAO and MitoTracker® Green (Invitrogen, Carlsbad, CA). Cells were grown to 50–75% confluency in a 6-well dish. The dyes were diluted in RPMI media (final concentrations: MitoTracker® Green = 0.5 µM, NAO = 20 nM) and exposed to the cells for 30 minutes at 37°C. Cells were washed with PBS, collected by scraping and analyzed using a FACStar flow cytometer (Becton-Dickinson Biosciences, San Jose, CA).

To measure mitochondrial membrane polarization, 1×10^6^ cells were suspended in 1 ml PBS and incubated with JC-1 dye (Invitrogen) at 0.2 µM for 30 minutes. Cells were pelleted by centrifugation and re-suspended in 500 µL PBS. The cells were analyzed by flow cytometry measuring both green and red fluorescence. Relative degrees of mitochondrial polarization were quantified by measuring the ratio of red-shifted JC-1 aggregates, which are favored under conditions of high membrane potential, and green-shifted monomers, which tend to predominate under conditions of low membrane potential [41]. Additionally, cells stained with MitoTracker® Deep Red FM (final concentration 0.5 µM) were used to assess both mass and membrane potential.

### PEG-Fusion Assay

5×10^5^ MycER cells expressing mito-GFP were co-plated with the same number of MycER cells expressing mito-DsRed onto glass coverslips in 12 well plates. The following day, the coverslips were incubated with DMEM+cycloheximide (CHX, 33 µg/ml, Sigma) for 30 min to inhibit de novo synthesis of fluorescent proteins. Next, coverslips were incubated in 0.5 ml PEG 1500 (Sigma) for 1 min at room temperature, washed with DMEM+CHX three times, and subsequently incubated in the same medium at 37°C and 5% CO_2_. One or two hours later, coverslips were fixed for 30 min with ice-cold 3.7% formaldehyde in PBS, stained with DAPI and mounted onto glass microscope slides. A minimum of 20 heterokaryons were observed using confocal fluorescence microscopy from which the percentage of co-localized fluorescence was calculated using CoLocalizer Pro software (CoLocaliztion Research Software; [70], [71]) and expressed as the percentage of mitochondrial fusion.

### Measurements of cellular OXPHOS and glycolysis

All measurements were performed with a Seahorse Bioscience XF24 Extracellular Flux Analyzer (Billirica, MA). Cells were plated at 20,000 cells/well onto Seahorse 24 well plates 12–18 hours prior to the assay. Immediately following the addition of fresh medium, O_2_ consumption rate (OCR) and proton production, expressed as the extracellular acidification rate (ECAR), were quantified to obtain baseline levels of these processes. The next measurement was performed following the blockade of complex V by 1 µM oligomycin (injection A). This causes a build-up of protons across the inner mitochondrial matrix with subsequent loss of electron flow. The addition of FCCP (final concentration 300 nM; injection B) causes the protons on the outside of the inner membrane to be carried across to the mitochondrial matrix. The addition of 2-deoxyglucose (2-DG) (final concentration 100 mM; injection C) inhibits glucose uptake, glycolysis, and the generation of acetyl coenzyme A from pyruvate for utilization as an initial substrate for the TCA cycle. Finally, the injection of rotenone (final concentration 1 µM; injection D) inhibits complex I, leading to cessation of both electron flow and oxygen consumption. Experiments were performed by simultaneously measuring three to five replicates of each cell line. Relative effects were expressed as areas under the curve measurements that were generated by the manufacturer's software.

### Measurement of ATP levels

ATP levels were measured using the ATPlite™ Luminescence Assay System (Perkin Elmer, Waltham, MA) according to manufacturer's instructions. 20,000–30,000 cells were grown overnight in 96-well plates and were exposed to 100 µl of medium with and without metabolic inhibitors at 37°C for the final 45 minutes prior to the assay. The concentrations of the inhibitors were the same as those used for the Seahorse assay described above.

The half-life of ATP was determined by first growing the cells as described above. Warm medium containing both oligomycin and 2-DG was then added and the cells were incubated at room temperature. At various points thereafter, lysis solution was added to stop the reaction. Luminescence was measured as described above and the exponential line equation was used to calculate the half-life.

### Preparation of mitochondria from fibroblasts

Approximately 10^7^ freshly harvested fibroblasts were re-suspended in 0.5 ml of ice cold buffer 25 mM Tris-HCl, pH 7.5; 100 mM KCl; 0.4 M sucrose containing protease inhibitor cocktail (Sigma-Aldrich) and disrupted in a cell homogenizer (Isobiotec, Heidelberg, Germany) for 10 strokes on ice. The resultant homogenate was clarified by centrifugation at 500× *g* for 10 min at 4°C, and the pellet was discarded. The mitochondria-rich supernatant was then centrifuged at 14,000× *g* for 15 min. The pellet was washed twice with buffer A and re-suspended in same buffer at a final protein concentration of 5 mg/ml. Samples were either analyzed immediately or stored at −80°C.

### Blue native gel electrophoresis (BNGE)

Eight mg of digitonin (MP Biomedicals, Solon, OH) was dissolved in 200 µl of 30 mM HEPES buffer, pH 7.4, 150 mM potassium acetate and 10% glycerol, heated at 95°C, then cooled on ice. One mg of mitochondria, isolated as described above, was lysed by the addition of digitonin to a final ratio of 1∶8 protein∶digitonin. Following incubation on ice for 20 min, a Coomassie blue solution (5% Coomassie blue G250 in 750 mM 6-aminocaproic acid) was added (1/20 v/v), and the mixture was centrifuged at 14,000× *g* for 20 min at 4°C. The supernatant was then directly loaded onto a 3–12% Native PAGE Novex Bis-Tris gel (Invitrogen), and subjected to electrophoresis at 80 V for 4 hours at 4°C in the buffer provided by the supplier. Following electrophoresis, gels were stained with Bio-Safe Coomassie G250 (Bio-Rad, Hercules, CA) for 30 min and exhaustively de-stained with water. Stained gels were scanned and the images analyzed for relative band density using AlphaEaseFC 2200 scanner and AlphaEaseFC software.

For confirmation of native complex identities, individual bands were excised from the above gels, incubated in Laemmli SDS-sample buffer (Bio Rad) for 30 min at room temperature and electrophoresed on a Criterion pre-cast gel (Bio Rad) and electrophoresed at 80 V for 2 hours followed by silver staining (Sigma-Aldrich).

### Complex I and V gel *in situ* activity stain

For Complex I activity measurements, the blue native gel was placed in 3–4 ml of 2 mM Tris-HCl, pH 7.4 buffer containing 2.5 mg/ml nitrotertrazolinum blue chloride and freshly added 0.1 mg/ml NADH. The gel was incubated at 37°C for 1–2 hours and then subjected to densitometric analysis. An average value from 3 gels was calculated. To quantify the ATPase activity of Complex V, gels were incubated in 3–4 ml of 34 mM Tris-glycine, pH 7.8, 14 mM MgSO_4_, 0.2% Pb(NO_3_)_2_ with freshly added 8 mM ATP for 3 hours at 37°C. Bands were quantified as for Complex I.

### Immunoblotting

Cells were grown to approximately 90% confluency under standard conditions and then harvested by trypsinization. After washing twice in PBS, cell pellets were lysed in SDS-PAGE lysis buffer without β-mercaptoethanol, and protein concentrations were quantified using a BCA protein determination kit (Pierce, Rockford, IL). 5 µg of total protein lysate from each cell line was then resolved on a 10% SDS-polyacrylamide gel and transferred to a PVDF membrane (Millipore, Inc., Bedford, NY) by electrophoretic transfer according to manufacturer's instructions (Bio-Rad). Immunoblotting was performed as previously described [72]. The antibodies and the concentration at which they were used are listed in Table S1. The blots were developed using chemiluminesence (Thermo Fisher Scientific, Rockford, IL).

## Supporting Information

Figure S1 **Western analysis for Myc expression.** 5 µg of whole cell lysates from *myc+/+*, *myc−/−* and *myc−/−*wtMyc cells were used to perform Western analysis with the 9e10 anti-Myc antibody. β-actin is used as a loading control.
(TIF)

Figure S2 **ECAR in rat fibroblasts.** Extracellular acidification rates (ECARs) were calculated concurrently with OCR. ECAR is a surrogate measure of glycolysis and is expressed as a function of time. Inhibitors were injected at the times indicated by the arrows (injections: A-oligomycin, B-FCCP, C-2-DG, D-rotenone). A typical experiment, performed in triplicate wells is shown. The experiment was repeated on at least three occasions in replicates of four with similar results.
(TIF)

Figure S3 **ATP half life.** ATP levels were measured for the *myc+/+* and *myc−/−*+wtMyc fibroblasts. The cells were incubated for the indicated times in the presence of 2-DG and oligomycin. A logarithmic curve was fit for each data set and the equation of the line was used to calculate the half life. Depicted is a representative experiment.
(TIF)

Table S1 **Antibodies used in this study.**
(PDF)

Video S1 **Mitochondrial structure changes following Myc deactivation in MycER cells.** MycER cells were observed under live confocal microscopy either prior to (left) or 24 h after the removal of 4-HT. Mitochondria were stained with MitoTracker Deep Red and 3-dimensional reconstructions of the cells were performed from a series of z-stacks. Note the loss of total mitochondria and the decrease in interconnectivity upon removal of 4-HT. Furthermore, note the preferential loss of perinuclear mitochondria.
(MOV)
