# Supplementary figures and images for: Mitochondrial Structure, Function and Dynamics Are Temporally Controlled by c-Myc
Source: PLoS One. 2012 May 21;7(5):e37699. doi: 10.1371/journal.pone.0037699 (PMC3357432; doi:10.1371/journal.pone.0037699)

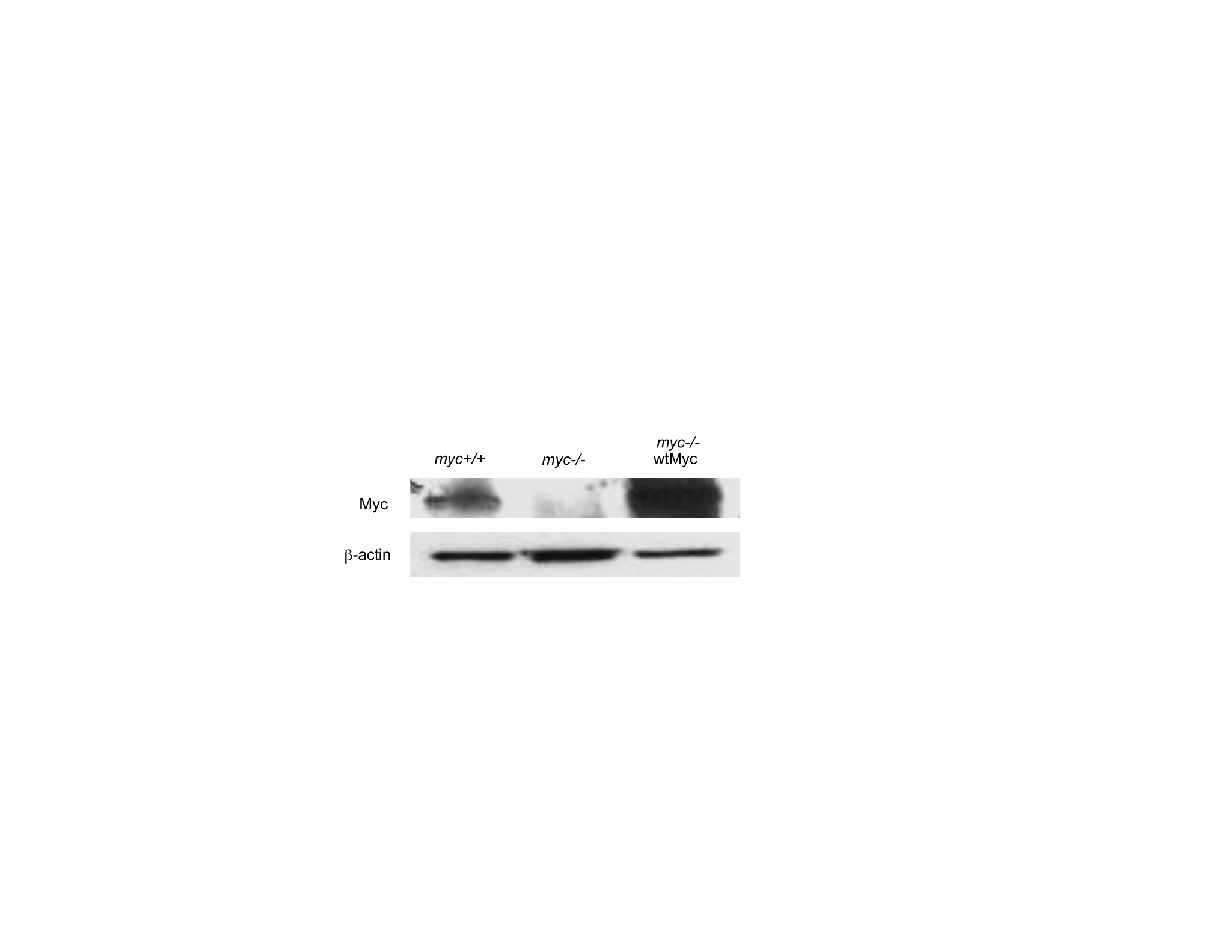

Supplement: Figure S1 — Western analysis for Myc expression. 5 µg of whole cell lysates from myc+/+, myc−/− and myc−/−wtMyc cells were used to perform Western analysis with the 9e10 anti-Myc antibody. β-actin is used as a loading control. (TIF) [file pone.0037699.s001.tif]

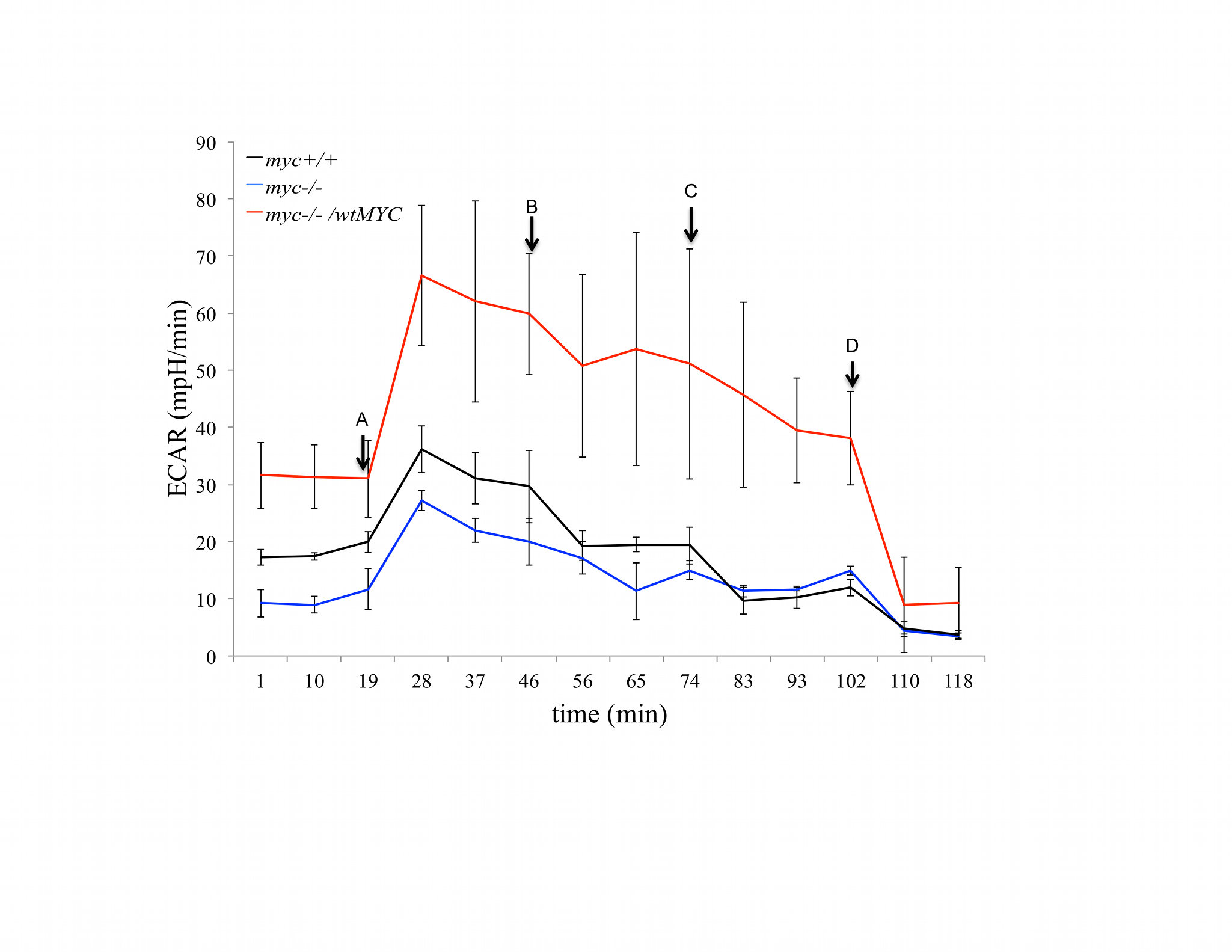

Supplement: Figure S2 — ECAR in rat fibroblasts. Extracellular acidification rates (ECARs) were calculated concurrently with OCR. ECAR is a surrogate measure of glycolysis and is expressed as a function of time. Inhibitors were injected at the times indicated by the arrows (injections: A-oligomycin, B-FCCP, C-2-DG, D-rotenone). A typical experiment, performed in triplicate wells is shown. The experiment was repeated on at least three occasions in replicates of four with similar results. (TIF) [file pone.0037699.s002.tif]

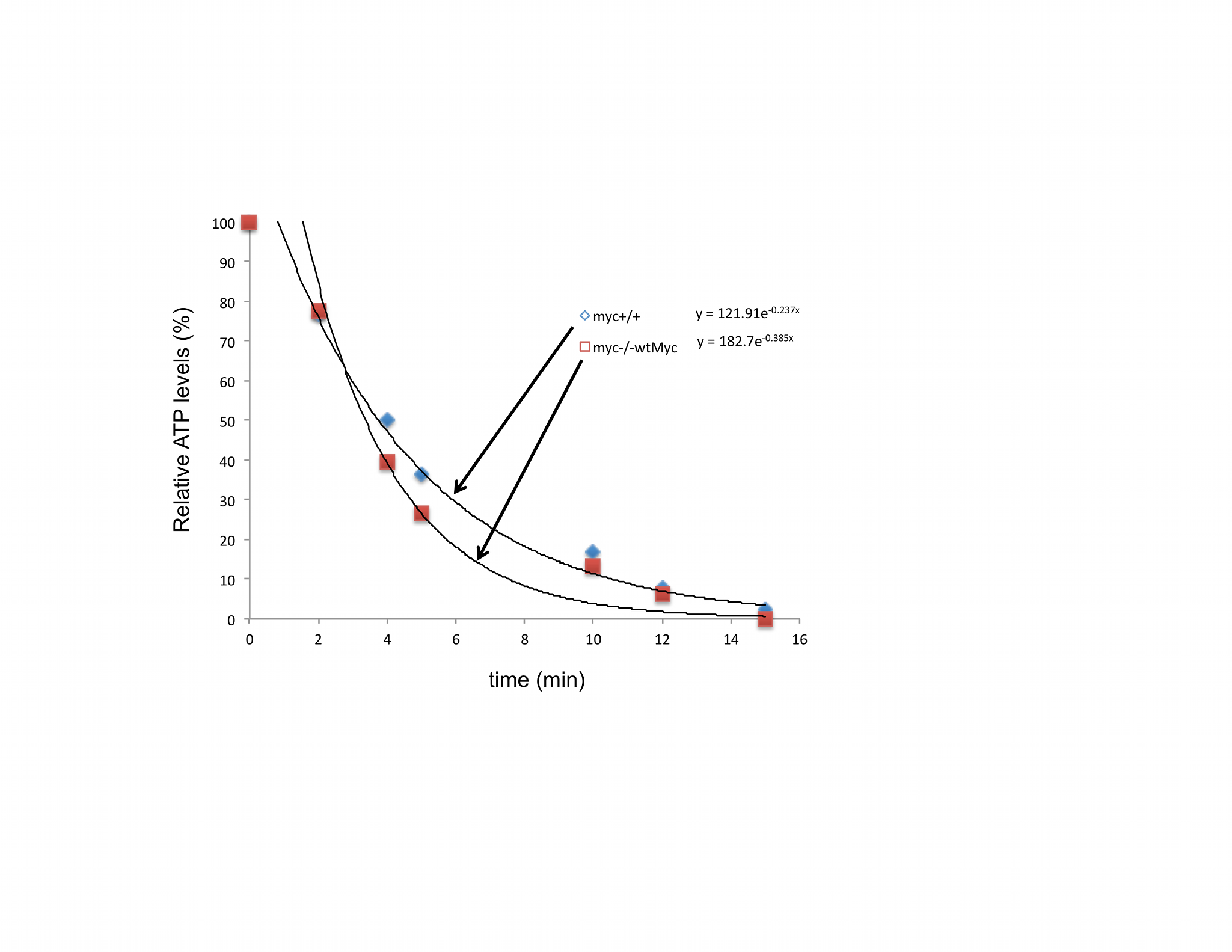

Supplement: Figure S3 — ATP half life. ATP levels were measured for the myc+/+ and myc−/−+wtMyc fibroblasts. The cells were incubated for the indicated times in the presence of 2-DG and oligomycin. A logarithmic curve was fit for each data set and the equation of the line was used to calculate the half life. Depicted is a representative experiment. (TIF) [file pone.0037699.s003.tif]
