# Supplementary material for: Mitochondrial Structure, Function and Dynamics Are Temporally Controlled by c-Myc
Source: PLoS One. 2012 May 21;7(5):e37699. doi: 10.1371/journal.pone.0037699 (PMC3357432; doi:10.1371/journal.pone.0037699)
Supplement: Table S1 — Antibodies used in this study. (PDF) [file pone.0037699.s004.pdf]

| Antibody | Dilution | Company | Catalog Number |
|---|---|---|---|
| Opa1 | 1:3000 | BD Biosciences; Franklin Lakes, NJ | 612606 |
| Mfn1 | 1:2000 | Santa Cruz Biotechnology; Santa Cruz, CA | sc-50330 |
| Mfn2 | 1:500 | Santa Cruz Biotechnology; Santa Cruz, CA | sc-100560 |
| Dlp1 | 1:3000 | BD Biosciences; Franklin Lakes, NJ | 611112 |
| Fis1 | 1:1500 | BioVision; Mountain View, CA | 3491-100 |
| β-actin | 1:1000 | Santa Cruz Biotechnology; Santa Cruz, CA | sc-81178 |
